# Metabolomics analysis of the effect of acidification on rhizosphere soil microecosystem of tea tree

**DOI:** 10.3389/fpls.2023.1137465

**Published:** 2023-02-24

**Authors:** Jianghua Ye, Yuhua Wang, Shaoxiong Lin, Yuchao Wang, Pengyuan Chen, Lei Hong, Xiaoli Jia, Jiaqian Kang, Zeyan Wu, Haibin Wang

**Affiliations:** ^1^ College of Tea and Food, Wuyi University, Wuyishan, China; ^2^ College of Life Science, Fujian Agriculture and Forestry University, Fuzhou, China; ^3^ College of Life Science, Longyan University, Longyan, China

**Keywords:** tea tree, acidification, soil physicochemical indexes, microorganism, soil metabolomics

## Abstract

Acidification can seriously affect the growth of tea trees and the yield and quality of tea leaves. In this study, we analyzed the effects of acidification on the physicochemical properties, microorganisms and metabolites of tea rhizosphere soils with different pH values, and the results showed that with the increase of soil pH, the organic matter content, cation exchange capacity, microbial biomass carbon, microbial biomass nitrogen, microbial respiration intensity, bacterial number and actinomyces number in tea rhizosphere soil all showed an increasing trend, while the fungi number decreased. The results of soil metabolite analysis showed that 2376, 2377 and 2359 metabolites were detected in tea rhizosphere soil with pH values of 3.29, 4.74 and 5.32, respectively, and the number of similar compounds reached 2331, accounting for more than 98%. The results of soil metabolite content analysis showed that with the increase of soil pH, the total contents of metabolite of tea rhizosphere soil increased significantly. The results of correlation analysis between physicochemical indexes of soil and microorganisms and soil metabolites showed that physicochemical indexes of soil and microorganisms were significantly correlated with 221 soil metabolites, among which 55 were significantly positively correlated and 166 were significantly negatively correlated. Based on correlation interaction network analysis, 59 characteristic compounds were obtained and divided into 22 categories, among which 7 categories compounds showed a significant increasing trend with the increase of soil pH, while the other 15 categories compounds showed the opposite trend. Based on the functional analysis of characteristic metabolites, this study found that with the increase of soil pH in tea rhizosphere, the diversity and number of soil microorganisms increased, and the cyclic ability of C and N of tea rhizosphere soil was enhanced, which in turn might lead to the enhancement of resistance of tea tree and promote the growth of tea tree.

## 1 Introduction

Tea tree (*Camellia sinensis*) is a crop with high economic value, which has an important role in promoting the development of agriculture. Tieguanyin is one of the top ten famous teas in China, and Tieguanyin tea tree is native to Anxi County, Quanzhou City, Fujian Province, China (117°36 ‘~ 118°17’ E, 24°50’ ~ 25°26’ N). Anxi County is the main tea producing county in China, with tea production ranking first in China for 8 consecutive years (From 2014 to 2021). Tea tree is an acidophilic crop, however, when the soil pH was lower than 4.0, the soil was severely acidified and the growth of tea tree and its tea yield and quality were significantly reduced (Lin et al., 2022b; Wang et al., 2022). In recent years, soil acidification in tea plantations in Anxi County has intensified and severely limited the development of tea industry. For example, in 2018, Wang et al. (2018) investigated the soil pH of 363 tea plantations in Anxi County and found that 37.67% of the soil pH of tea plantations was lower than 4.5, indicating acidification of tea plantation soil. In 2021, Yang (2021) investigated the soil pH of 5285 tea plantations in Anxi County and found that 68.44% of the tea plantations had a soil pH lower than 4.5, and 28.38% of tea plantations had a soil pH below 4.0. It can be seen that in only 3 years, soil acidification area of tea plantations in Anxi County has expanded nearly one time, which has caused serious impact on the development of tea industry in Anxi County.

In recent years, many scholars have devoted themselves to studying the effects of soil acidification on the growth of tea tree and the rhizosphere soil microecosystem of tea tree. It has been found that the key reason for the reduction of tea yield and quality due to soil acidification was the imbalance of soil microecosystem, which in turn led to the reduction of soil nutrient cycling ability and the reduction of absorption and utilization ability of soil nutrients by tea trees (Han et al., 2022; Yang et al., 2022; Ye et al., 2023). For example, Lin et al. (2022a) used genomics to analyze the soil nitrogen conversion capacity of tea plantations with different soil pH and found that as soil pH decreased, soil microbial diversity decreased, soil nitrogen conversion capacity decreased, and tea tree nitrogen absorption capacity decreased. Wang et al. (2020b) used soil proteomics to analyze the effect of acidification on microbial diversity in tea rhizosphere soil and found that with the decrease of soil pH, protein expression related to microbial reproduction, carbon, nitrogen and sulfur cycles in tea rhizosphere soil decreased, and microbial diversity decreased. It could be seen that the key to the effect of soil acidification on the growth of tea tree was the change of soil micro-ecosystem. Soil is the carrier of tea tree growth, and the root system is the key tissue connecting tea tree and soil. The root system of tea tree perceived changes in the external environment by releasing root secretions to interacting with substances in the soil, affecting changes in the number and types of soil microorganisms, and thus changing soil nutrient cycling (Shen and Lin, 2021; Wang et al., 2021; Tan et al., 2022). Therefore, an in-depth analysis of rhizosphere microecological changes from the perspective of soil metabolites was of great significance for further revealing the effects of acidification on the rhizosphere ecosystem of tea tree.

Soil metabolomics is a large-scale study of low-molecular-weight organic compounds in soil, and it provides an important means to reveal complex molecular networks and metabolic pathways in soil microbial communities and to evaluate soil function (Withers et al., 2020). In recent years, many scholars have used soil metabolomics to analyze the effects of environmental factors on soil microbial diversity and soil function, and a series of important research results have been obtained (Brown et al., 2021; Liang et al., 2021; Sun et al., 2022a; Wu et al., 2022). However, few studies have been reported using soil metabolomics to analyze the effects of acidification on microbial diversity and function in tea rhizosphere soil. This study is of great significance for further revealing the metabolism of soil microorganisms in tea rhizosphere and soil function caused by acidification. Therefore, in this study, tea plantations with different degrees of soil acidification were used as research objects to collect rhizosphere soil of tea trees, and on the one hand, soil physicochemical and microbial indexes were determined, and on the other hand, soil metabolites were analyzed using non-targeted metabolomics tools. On this basis, the relationship between soil physicochemical indexes, microorganisms and soil metabolites was further analyzed, in order to lay a certain foundation for further revealing the effects of acidification on microbial diversity and function of tea rhizosphere soil. This study will provide an important theoretical basis for soil remediation in acidified tea plantations.

## 2 Materials and methods

### 2.1 Field experiment and sample collection

Tieguanyin is one of the top ten famous teas in China, and Anxi County, Quanzhou City, Fujian Province, China, is the origin of Tieguanyin tea tree. The county is located at 117°36 ‘~ 118°17’ E, 24°50’ ~ 25°26’ N, with an average altitude of 600 m, annual average rainfall of 1800 mm, annual average relative humidity of 80% and annual average temperature of 18°C. On the basis of our previous studies (Wang et al., 2022), three adjacent tea plantations (P1, P2, P3) in Longjuan Township, Anxi County, Quanzhou City, Fujian Province (117°93 ‘E, 24°97’ N), were selected as research objects in this study, where the soil in P1 tea plantation was seriously acidified (pH < 4.0), the soil in P2 tea plantation was suitable for tea tree planting (4.5 < pH < 5.0), and the soil in P3 tea plantation was the most suitable for tea tree planting (5.0 < pH < 5.5). The tea tree variety planted in all three tea plantations was Tieguanyin and the age of the tea trees was seven. In May 2022, rhizosphere soil of tea trees from different tea plantations was collected for the determination of soil metabolites, pH value, organic matter content, cation exchange capacity, microbial biomass carbon, microbial biomass nitrogen, microbial respiration intensity, bacterial number, fungal number and actinomyces number. Three replicate samples were collected from rhizosphere soil of tea trees. The sampling method of tea rhizosphere soil was to remove the fallen leaves on the surface, gently digged out the tea tree, took out the tea tree root system, and shook off the soil attached to the root system surface, that was, the tea rhizosphere soil.

### 2.2 Determination of pH value, organic matter content and cation exchange capacity in soil

Soil pH was measured with a pH meter (PB-10, Sartorius) at a soil to water ratio of 1:2.5, and 5 replicates were used for each sample (Ye et al., 2022). Soil organic matter content was determined by oxidation with potassium dichromate and external heating, and soil cation exchange capacity was determined by neutral ammonium acetate (Wang et al., 2020a).

### 2.3 Determination of soil microbial biomass carbon, nitrogen and respiration intensity

Soil microbial biomass carbon and nitrogen were determined by the chloroform fumigation-extraction method with 3 replications to each sample (Zhang et al., 2021). Microbial biomass carbon was calculated using the formula Ec/Kc, where Ec is (Fumigated organic carbon) – (Unfumigated organic carbon), and Kc is 0.38. Microbial biomass nitrogen was calculated using the formula (total nitrogen in fumigated soil- total nitrogen in no fumigated soil)/0.54. Soil microbial respiration intensity (mg CO_2_/kg·h) was measured using the alkali absorption method, based on the amount of CO_2_ released from soil, per hour per kilogram (Zhang et al., 2021).

### 2.4 Quantitative analysis of soil bacteria, fungi and actinomyces by *q*RT-PCR

The total DNA of soil microorganisms was extracted using Bio 101 FastDNA SPIN Kit (Bio 101, Inc., USA) at a dosage of 0.5 g fresh soil. DNA was detected by 1% agarose gel electrophoresis, and purified using gel recovery kit (TianGen Biotech Co., Ltd). The purified DNA was used for quantitative *q*RT-PCR analysis of bacteria, fungi and actinomyces. The quantitative analysis method of *q*RT-PCR was carried out according to Chen et al. (2021), as follows.

The primers used for quantitative analysis of bacterial were F27 5’-AGAGTTTGATCMTGCCTCAG-3’, R1492 5’-TACHHYTACCTTGTTACGACTT-3’. The PCR reaction system was 25 μL, including 0.5 μL of template DNA, 12.5 μL of SYBR Premix Ex Taq (TaKaRa Biotechnology), 1 μL of each primers, and the rest was ddH_2_O; the PCR reaction procedures was 94 °C pre-denaturation for 4 min, 94 °C for 1 min, 55 °C for 1 min, 72 °C for 1 min, and fluorescence signals were collected for 30 cycles.

The primers used for quantitative analysis of fungi were 5.8S 5’-CGCTGCGTTCTTCATCG-3’, ITSIF 5’-CTTGGTCATTTAGAGGAAGTAA-3’. The PCR reaction system was 25 μL, including 1 μL of template DNA, 12.5 μL of SYBR Premix Ex Taq (TaKaRa Biotechnology), 1 μL of each primer, and the rest was ddH_2_O.The PCR reaction procedure was 95 °C pre-denaturation for 5 s, 94 °C for 30 s, 53 °C for 30 s, 72 °C for 30 s, fluorescence signals were collected for 40 cycles.

The primers used for quantitative analysis of actinomyces were Act920F 5’-TACGGCCGCAAGGCTA-3’, Act1200R 5’-TCRTCCCCACCTTCCTCCG-3’. The PCR reaction system was 25 μL, including 1 μL of template DNA, 12.5 μL of SYBR Premix Ex Taq (TaKaRa Biotechnology), 1 μL of each primer, and the rest was ddH_2_O; the PCR reaction procedures was, predenaturation at 95°C for 10 min, 95°C for 15 s, 65°C for 30 s, 72°C for 15 s, fluorescence signals were collected, and 40 cycles were performed.

### 2.5 Determination of soil metabolites

#### 2.5.1 Sample preparation and extraction

The non-targeted metabolomics method was used to analyze the metabolites in the rhizosphere soil of tea tree. This was done by taking 50 mg of soil samples and homogenizing them with 500 μL of ice-cold methanol/water (70%, v/v), respectively. The mixtures were homogenized at 30 Hz for 2 min. After homogenization, the mixture was shaken for 5 min and incubated on ice for 15 min, and centrifuged at 12,000 rpm and 4 °C for 10 min and suck 400 μL of supernatant into another centrifuge tube. 500 mL of ethyl acetate/methanol (V, 1:3) was added to the original centrifuge tube, oscillated for 5 min and incubated on ice for 15 min, centrifuged at 12,000 rpm and 4 °C for 10 min, and then took 400 mL of supernatant. The two supernatants were combined and concentrated. Then 100 μL of 70% methanol water was added into the dried product for 3min of ultrasonication. Finally, the product was centrifuged at 12,000 rpm and 4 °C for 3 min, and 60 μL of supernatant was aspirated for LC-MS/MS analysis.

#### 2.5.2 LC-MS/MS conditions

All samples were acquired by the LC-MS/MS system followed machine instructions. Analytical conditions of UPLC were as follows: column, Waters ACQUITY UPLC HSS T3 C18 (1.8 µm, 2.1 mm × 100 mm); column temperature, 40 °C; flow rate, 0.35 mL/min; injection volume, 2 μL; solvent system, water (0.04% acetic acid): acetonitrile (0.04% acetic acid); gradient program of positive ion, 95:5 V/V at 0 min, 79:21 V/V at 3.0 min, 50:50 V/V at 5.0 min, 30:70 V/V at 9.0 min, 5:95 V/V at 10.0 min, 95:5 V/V at 14.0 min; gradient program of negative ion, 95:5 V/V at 0 min, 79:21 V/V at 3.0 min, 50:50 V/V at 5.0 min, 30:70 V/V at 9.0 min, 5:95 V/V at 10.0 min, 95:5 V/V at 14.0 min. In MS analysis, positive ion mode and negative ion mode of electrospray ion source were adopted. The positive ion conditions were: voltage 250 V, gas flow 8 L/min, fragmetor 135 V, gas temperature 325 °C, sheath temperature 325 °C, sheath flow 11 L/min, and nebulizer 40 psi. The negative ion conditions were: voltage 1500 V, gas flow 8 L/min, fragmetor 135 V, gas temperature 325 °C, sheath temperature 325 °C, sheath flow 11 L/min, and nebulizer 40 psi.

#### 2.5.3 Data analysis

The original data file obtained by LC-MS analysis was firstly converted into mzML format by Proteowizard software. Peak extraction, alignment and retention time correction were performed by XCMS program. The “SVR” method was used to correct the peak area. Filter the peaks with deletion rate > 50% in each group of samples. After that, metabolic identification information was obtained by searching the laboratory’s self-built database and integrating the public database and metDNA.

### 2.6 Statistical analysis

Excel 2017 software was used to calculate the average value and variance of data, Rstudio 3.3 software was used to make Cloud and rain map, correlation matrix maps, violin chart, box chart, principal component maps, venn diagram, and bubble map; Heml 1.0 software was used to make heat maps; Cytoscape_v3.9.1 software was used to make the correlation analysis chart.

## 3 Results and discussion

### 3.1 Analysis of soil and microbiological physicochemical indexes in tea rhizosphere

**Figure 1:**
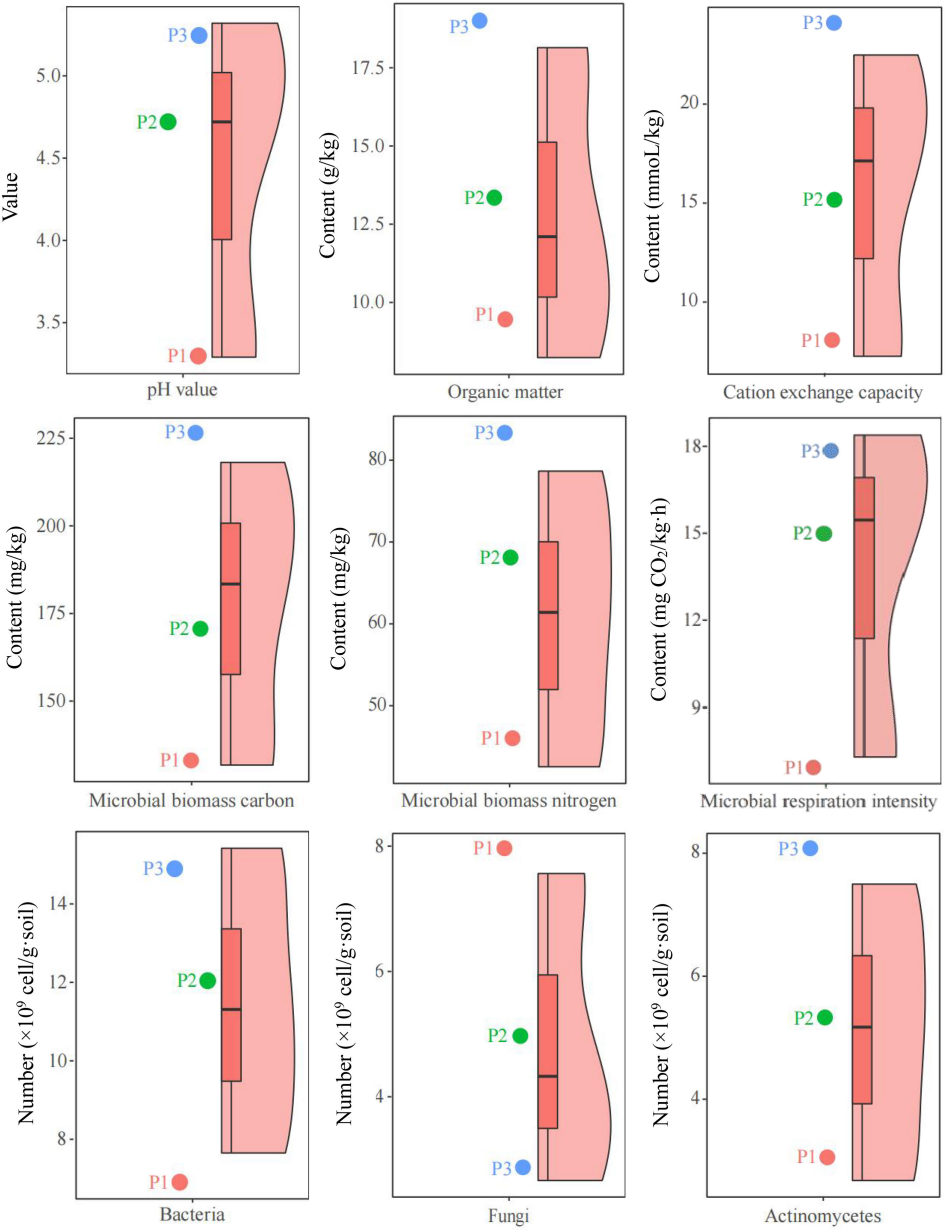
Analysis of soil and microbiological physicochemical indexes of different samples. P1: Tea plantation with severely acidified soils (pH 3.29); P2: Tea plantation with soils being suitable for tea tree planting (pH 4.74); P3: Tea plantation with soils being most suitable for tea tree planting (pH 5.32).

Tea tree is an acidophilic crop, however, changes in soil pH can affect the growth of tea tree (Wang et al., 2022). The results of this study showed (**Figure 1**) that for the 3 tea plantations (P1, P2, P3) selected in this study, the pH value of tea tree rhizosphere soil in the P1 tea plantation was 3.29, and the soil was seriously acidified and unsuitable for tea tree planting; in P2 tea plantation, pH value of tea tree rhizosphere soil was 4.74, which was suitable for tea tree planting; In P3 tea garden, pH value of tea tree rhizosphere soil was 5.32, and the soil was the most suitable for tea tree planting. On this basis, this study further analyzed the physicochemical and microbial indexes of soils with different pH values, and the results showed (**Figure 1**) that with the increase of soil pH (3.29~5.32), organic matter content (8.32 ~ 17.96 g/kg), cation exchange capacity (7.26 ~ 22.48 mmoL/kg), microbial biomass carbon (131.25 ~ 218.52 mg/kg), microbial biomass nitrogen (42.37 ~ 78.49 mg/kg), microbial respiration intensity (7.16 ~ 18.25 mg CO_2_/kg·h), bacterial number (7.65 ~ 15.42 ×10^9^ cell/g·soil), actinomyces number (2.65 ~ 7.52 ×10^9^ cell/g·soil) all showed an increasing trend, while the number of fungi (7.43~2.48 ×10^9^ cell/g·soil) decreased.

**Figure 2:**
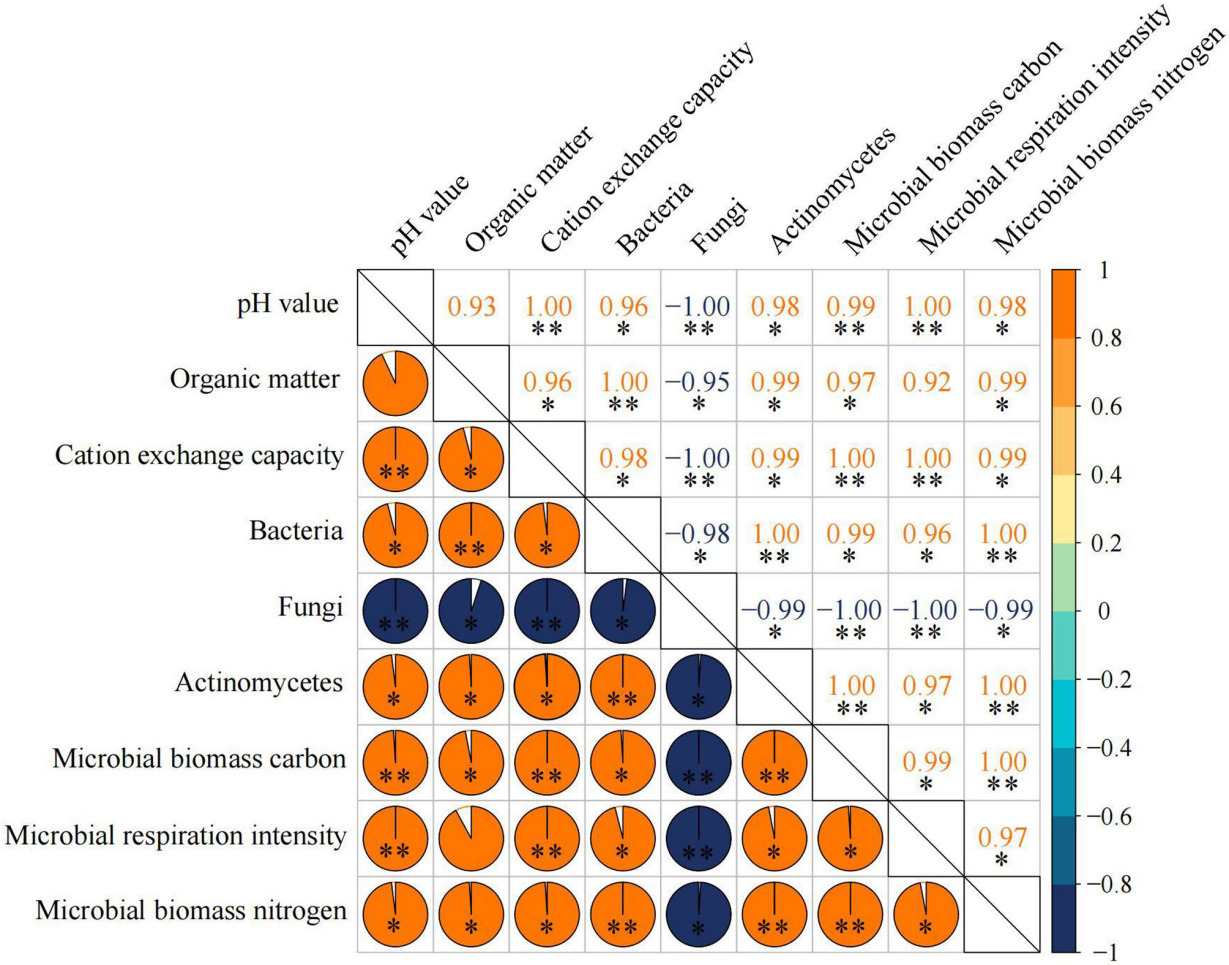
Correlation matrix analysis of different physicochemical indexes of soil and microorganism. * and ** represent a significant level at P < 0.05 and at P < 0.01, respectively.

The results of correlation matrix analysis showed (**Figure 2**) that there were significant positive correlations between soil pH, organic matter, cation exchange capacity, microbial biomass carbon, microbial biomass nitrogen, microbial respiration intensity, bacteria and actinomyces in tea rhizosphere soil, and they were significantly negatively correlated with fungi. It was reported that the increase of soil pH was beneficial to improve the cation exchange capacity of soil and accelerate the soil nutrient cycle, which in turn improved the soil microbial reproduction ability. However, after soil acidification, the number of soil bacteria and actinomyces decreased, the number of fungi increased, soil texture deteriorated, and plant growth was hindered (Wang et al., 2020b; Tayyab et al., 2021; Ding et al., 2022). It could be seen that with the increase of soil pH, the content of organic matter in soil increased, the cation exchange capacity increased, and the number of soil microorganisms increased, which was conducive to the improvement of soil nutrient cycling ability.

### 3.2 The number and content analysis of metabolites in rhizosphere soil of tea tree

**Figure 3:**
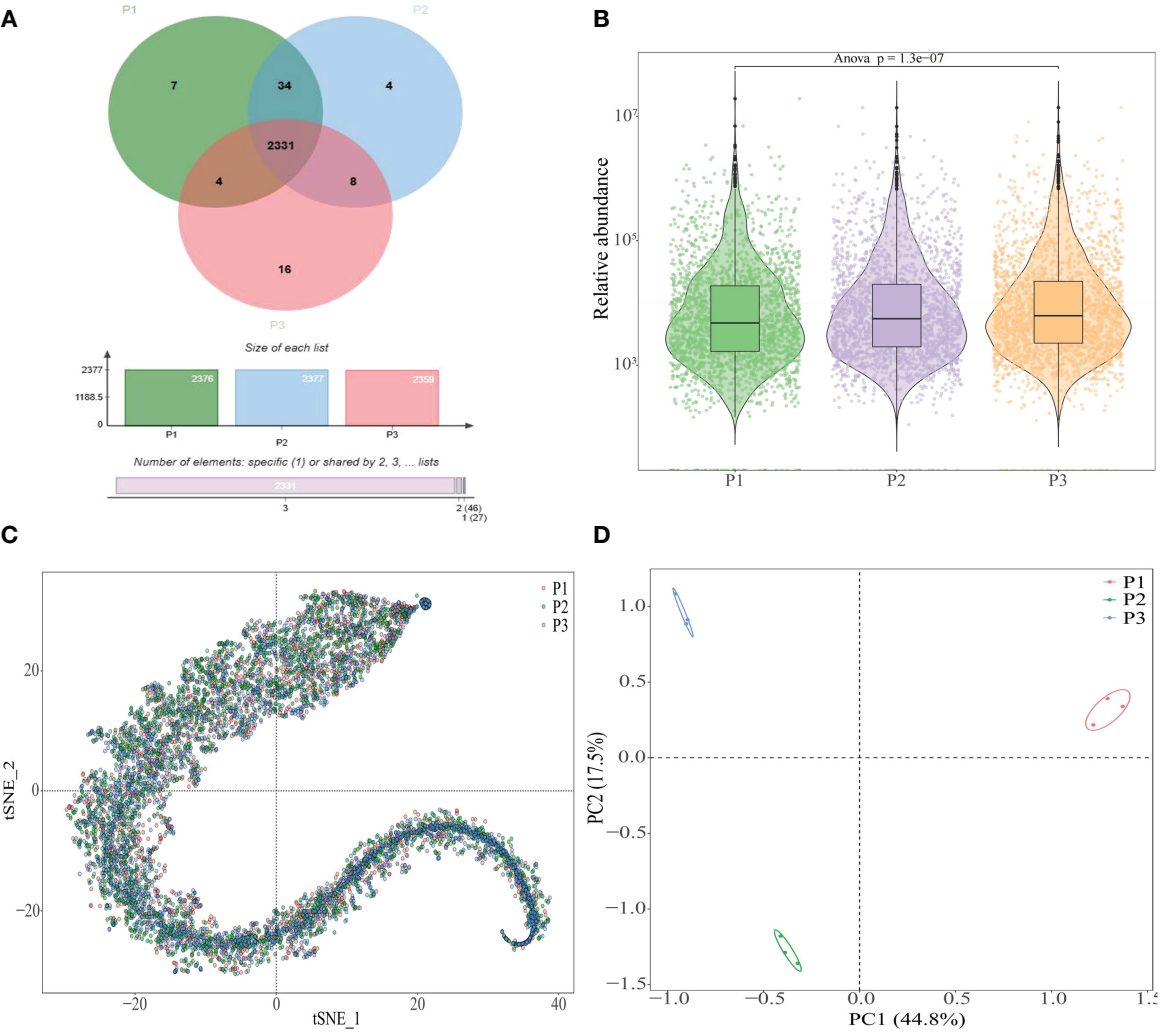
Analysis of metabolites in rhizosphere soil of tea tree with different pH values. P1: Tea plantation with severely acidified soil (pH 3.29); P2: Tea plantation with soils being suitable for tea tree planting (pH 4.74); P3: Tea plantation with soils being most suitable for tea tree planting (pH 5.32); **(A)** Venn diagram analysis of metabolites in rhizosphere soil of tea tree with different pH values; **(B)** Analysis of metabolites content in rhizosphere soil of tea tree with different pH values; **(C)** tSNE analysis of metabolites in rhizosphere soil of tea tree with different pH values; **(D)** Principal component analysis of metabolites in rhizosphere soil of tea tree with different pH values.

In order to adapt to environmental changes during the growth, plants regularly modified the rhizosphere microecological environment by regulating the type or amount of their root exudes to ensure their own growth (Barra Caracciolo and Terenzi, 2021; Bi et al., 2022). pH value was an environmental factor that caused soil acidification and significant changes in soil physiological and biochemical characteristics when the soil pH value was too low, and plants regularly modified the rhizosphere soil environment by changing the metabolites secreted by the roots to adapt to their growth (Griebenow et al., 2022; Zhang et al., 2022). In this study, the number and types of metabolites in rhizosphere soil of tea tree with different pH were analyzed, and the results showed (**Figure 3A**) that 2376, 2377 and 2359 metabolites were detected in rhizosphere soil of tea tree with pH values of 3.29, 4.74 and 5.32, respectively, of which 2331 kinds of similar compounds were detected, accounting for more than 98%. The analysis results of metabolite content in rhizosphere soil showed that (**Figure 3B**) the total metabolite content in rhizosphere soil of tea tree showed a significant increase trend with the increase of soil pH. Further analysis showed that although the types of metabolites in rhizosphere soil of tea tree with different pH were basically similar, there were certain differences in the content (**Figure 3C**). The results of the principal component analysis showed (**Figure 3D**) that metabolites detected in rhizosphere soil of tea tree with different pH values could effectively distinguish different samples. It could be seen that the effect of soil pH has little influence on the number and type of metabolites in the rhizosphere soil of tea tree, but has significant influence on their contents.

### 3.3 Classification analysis of metabolites in rhizosphere soil of tea tree

**Figure 4:**
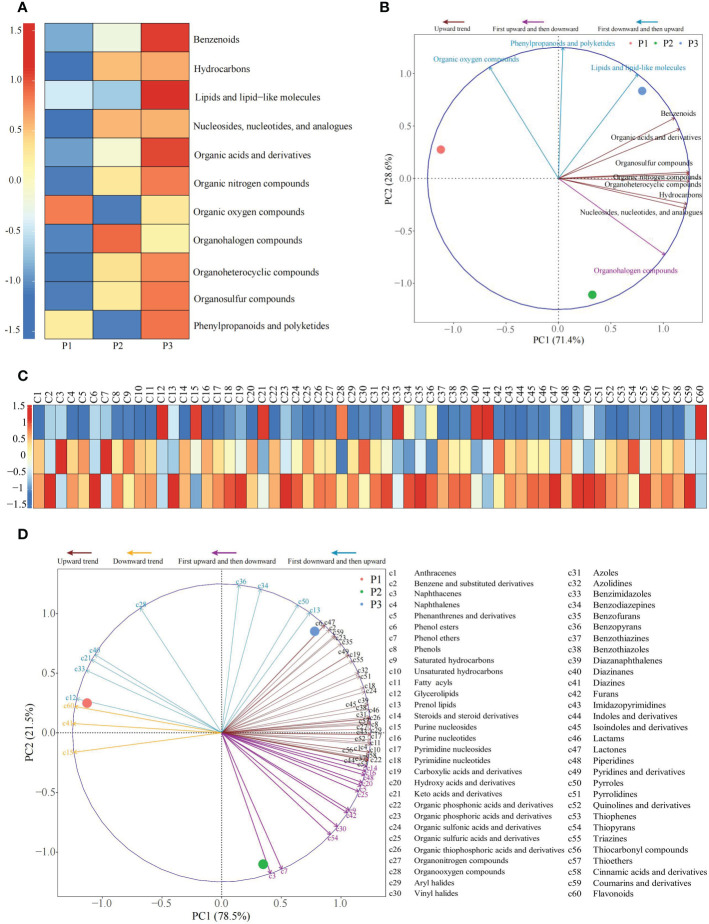
Principal component analysis of primary and secondary classification of metabolites in rhizosphere soil of tea tree with different pH values. P1: Tea plantation with severely acidified soil (pH 3.29); P2: Tea plantation with soils being suitable for tea tree planting (pH 4.74); P3: Tea plantation with soils being most suitable for tea tree planting (pH 5.32); **(A)** Heat map of the content of the primary classification of metabolites; **(B)** Principal component analysis for primary classification of metabolites; **(C)** Heat map of the content of the secondary classification of metabolites; **(D)** Principal component analysis of secondary classification of metabolites.

The results of the analysis of the classification of metabolites and their contents in rhizosphere soil of tea tree (**Figure 4A**) showed that the primary classification could be divided into 11 categories, among which 7 categories compounds (Benzenoids, Hydrocarbons, Organic acids and derivatives, Organic nitrogen compounds, Organoheterocyclic compounds, Organosulfur compounds, Nucleosides, nucleotides, and analogues), showed an upward trend with the increase of soil pH, 3 categories compounds (Lipids and lipid-like molecules, Organic oxygen compounds, Phenylpropanoids and polyketides) decreased first and then increased, and only 1 category compound (Organohalogen compounds) increased first and then decreased. The results of the principal component analysis of the primary classification of soil metabolites showed (**Figure 4B**) that 11 categories compounds could effectively distinguish three different soil samples, among which 3 categories compounds with a decreasing trend followed by an increasing trend could effectively distinguish soil samples with pH values of 3.29 and 5.32, while 7 categories compounds with an upward trend and the 1 category compound with an upward trend followed by a downward trend could effectively distinguish soil samples with pH values of 4.74 and 5.32.

The secondary classification of metabolites in rhizosphere soil of tea tree was further analyzed, and the results showed (**Figure 4C**) that the secondary classification contained 60 categories, among which, 36 categories compounds showed an upward trend, 3 categories compounds showed a downward trend, 12 categories compounds showed an upward trend and then a downward trend, and 9 categories compounds showed a downward trend and then an upward trend as the soil pH increased. The results of the principal component analysis of the secondary classification of soil metabolites showed (**Figure 4D**) that 60 categories compounds could effectively distinguish three different soil samples, among which 3 categories compounds with a declining trend could effectively distinguish soil samples with pH values of 3.29 and 4.74, 9 categories compounds with a tendency of first decreasing and then increasing could effectively distinguish soil samples with pH values of 3.29 and 5.32, while 36 categories compounds with an increasing trend and 12 categories compounds with first rising and then falling trend could effectively distinguish soil samples with pH values of 4.74 and 5.32. It could be seen that the change of soil pH significantly changed the content of metabolites in tea rhizosphere soil, and there are significant differences between soils with different pH values.

### 3.4 Interaction network analysis of physicochemical indexes and metabolites in rhizosphere soil of tea tree

**Figure 5:**
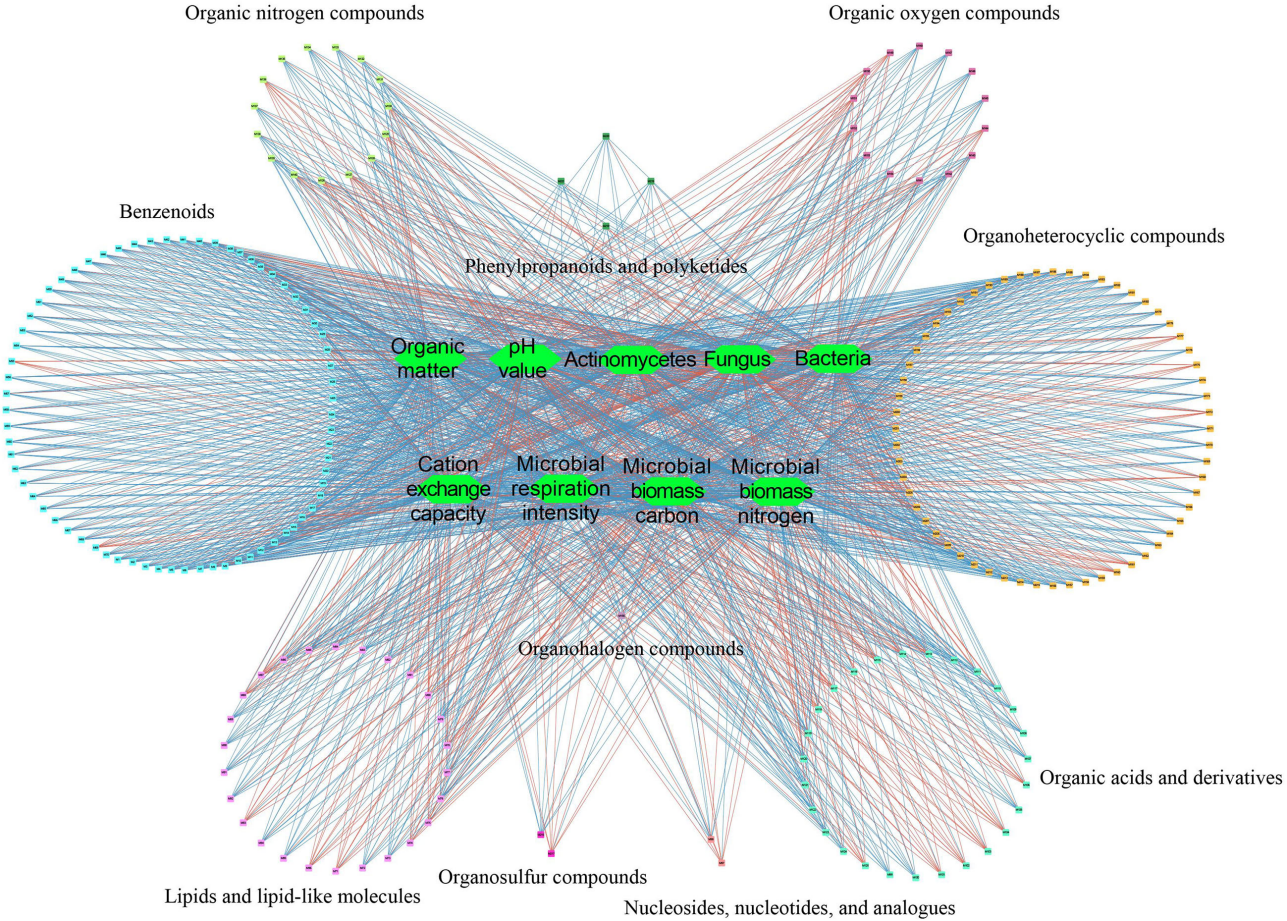
Interaction network analysis of physicochemical indexes and metabolites in tea rhizosphere soil. Significant positive correlation; Significant negative correlation.

The correlation analysis between soil and microbial physicochemical indexes and soil metabolites showed (**Figure 5**) that soil and microbial physicochemical indexes were significantly correlated with 221 soil metabolites, including 70 benzenoids, 26 lipids and lipid-like molecules, 2 nucleosides, nucleotides, and analogues, 27 organic acids and derivatives, 15 organic nitrogen compounds, 14 organic oxygen compounds, 1 organohalogen compounds, 60 organoheterocyclic compounds, 2 organosulfur compounds, 4 phenylpropanoids and polyketides. Further analysis showed that 55 soil metabolites were significantly positively correlated with soil pH, organic matter, cation exchange capacity, microbial biomass carbon, microbial biomass nitrogen, microbial respiration intensity, bacteria and actinomyces, while the remaining 166 soil metabolites were significantly negatively correlated. Secondly, fungi were significantly negatively correlated with the 55 soil metabolites mentioned before, and were significantly positively correlated with the remaining 166 soil metabolites. It could be seen that there was a close relationship between soil and microbial physicochemical indexes and soil metabolites.

### 3.5 Analysis of characteristic compounds in rhizosphere soil of tea tree

**Figure 6:**
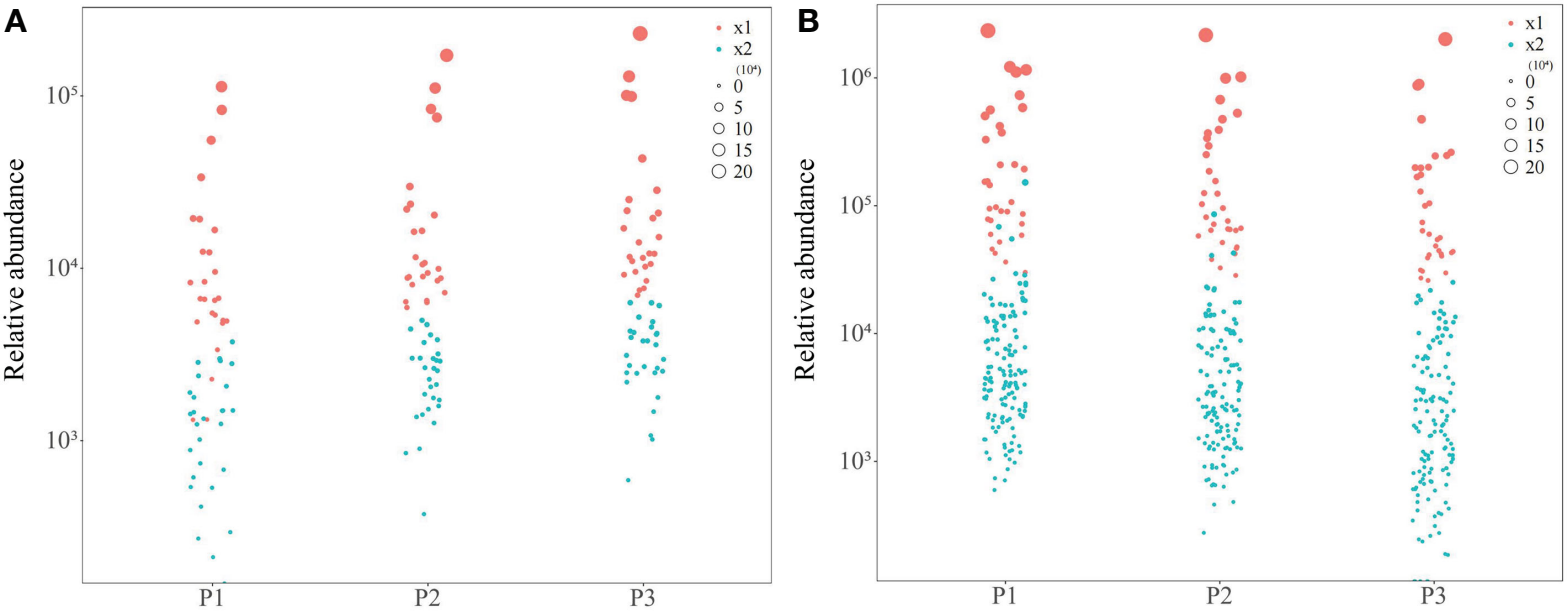
Bubble map analysis of tea tree rhizosphere soil characteristic metabolites screening at different pH. P1: Tea plantation with severely acidified soil (pH 3.29); P2: Tea plantion with soils being suitable for tea tree planting (pH 4.74); P3: Tea plantation with soils being most suitable for tea tree planting (pH 5.32); x1: Characteristic metabolites; x2: Non-characteristic metabolites; **(A)** Bubble map of metabolites positively correlated with physicochemical indexes of rhizosphere soil of tea tree; **(B)** Bubble map of metabolites negatively correlated with physicochemical indexes of rhizosphere soil of tea tree.

On the basis of the above correlation interaction network analysis, bubble map characteristics were analyzed according to the content of 55 soil metabolites with significant positive correlation, and 26 characteristic compounds were obtained with more than 90% of the total number of positively correlated compounds (**Figure 6A**). According to the 166 soil metabolites with significant negative correlation, bubble map characteristics were performed with their contents, and 33 characteristic compounds were obtained, accounting for more than 90% of the total number of negatively correlated compounds (**Figure 6B**).

**Figure 7:**
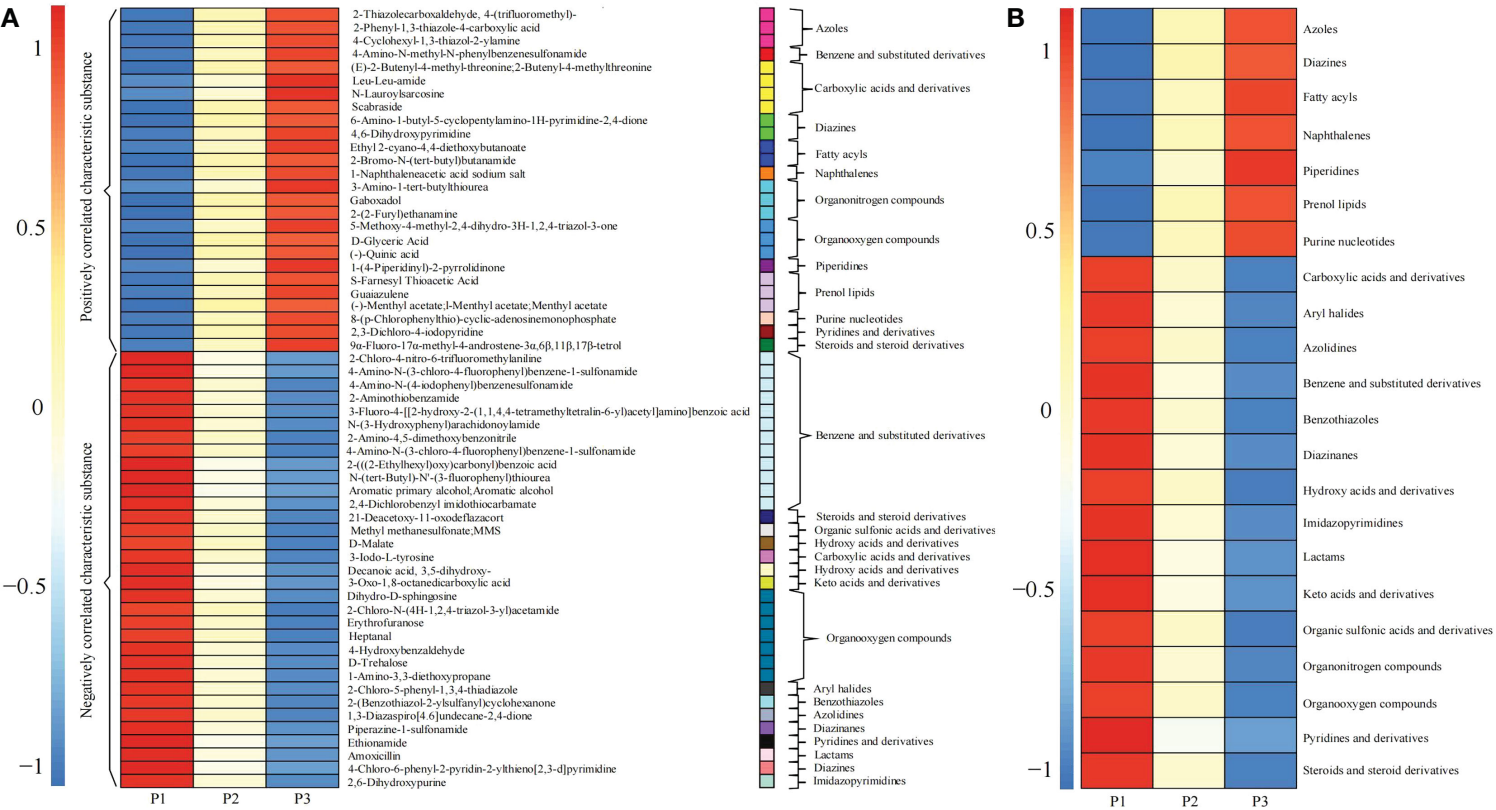
Content and classification of 59 kinds of characteristic compounds in rhizosphere soil of tea tree at different pH values. P1: Tea plantation with severely acidified soil (pH 3.29); P2: Tea plantation with soils being suitable for tea tree planting (pH 4.74); P3: Tea plantation with soils being most suitable for tea tree planting (pH 5.32); **(A)** Heat map analysis of 59 characteristic compounds; **(B)** Heat map analysis of 59 characteristic compounds after classification.

A total of 59 characteristic compounds were obtained for classification analysis (**Figure 7A**), and it was found that 59 characteristic compounds could be divided into 22 categories, among which 7 categories compounds showed a significant increasing trend with the increase of soil pH value (**Figure 7B**), namely azoles, diazines, fatty acyls, naphthalenes, piperidines, prenol lipids and purine nucleotides. It has been reported that diazines and purine nucleotides compounds, as precursors of nucleic acid biosynthesis, were raw materials required for microbial reproduction, and their derivatives had strong inhibitory and hindering effects on the reproduction of a variety of pathogens (Garg et al., 2020; Bertola et al., 2021; Sepúlveda et al., 2022). Fatty acyls compounds were the component of soil microbial cell membrane, and the increase of soil pH was beneficial to the accumulation of fatty acyls, which in turn improved the reproductive ability of microorganisms (Warren, 2021). Naphthalenes and piperidines compounds could effectively inhibit pathogen reproduction, control pests and diseases, and increase the number and diversity of beneficial soil microorganisms (Lan et al., 2019; Boadi et al., 2021; León-Morales et al., 2022). Azoles compounds were a class of antifungal compounds that played an important role in the control of plant pathogens (Rhijn et al., 2021; Sun et al., 2022b). Prenol lipids were natural antibiotics, which were beneficial to alleviate the damage caused by adversity stress and improved the adaptability of plants to the environment (Baczewska-Dąbrowska et al., 2022). It could be seen that with the increase of tea tree rhizosphere soil pH, the content of prenol lipids compounds in rhizosphere soil of tea tree increased, and the ability of tea tree to resist the stress of external environment increased, which in turn improved the adaptability of tea tree to the environment; in addition, the content of naphthalenes, piperidines and azoles compounds increased, which reduced the propagation of pathogenic microorganisms in soil, improved the soil microecological environment and enhanced the ability of tea tree to resist pests and diseases; the increase in the content of diazines, purine nucleotides, fatty acyls compounds provided raw materials for the propagation of beneficial microorganisms in the rhizosphere soil of tea tree, and increased the diversity and number of soil microorganisms.

Secondly, this study found that after 59 characteristic compounds were divided into 22 categories, among which 15 categories showed a significant downward trend (**Figure 7B**), namely carboxylic acids and derivatives, aryl halides, azolidines, benzene and substituted derivatives, benzothiazoles, diazinanes, hydroxy acids and derivatives, imidazopyrimidines, keto acids and derivatives derivatives, lactams, organic sulfonic acids and derivatives, organonitrogen compounds, organooxygen compounds, pyridines and derivatives, steroids and steroid derivatives. It was reported that aryl halides (Lei et al., 2021), benzothiazoles (Liao et al., 2018), lactams (Zheng et al., 2021), azolidines (Rajput and Mishra, 2019), pyridines and derivatives (Eryılmaz et al., 2020) compounds not only inhibited plant growth, especially plant roots, but also inhibited soil microbial propagation and reduced soil microbial diversity. Steroids and steroid derivatives reduced the diversity and quantity of soil bacteria (Nakayasu et al., 2021). Imidazopyrimidines were organic compounds containing pyrimidines whose decomposition facilitated to increase the amount of pyrimidines in soil, which in turn promoted microbial reproduction (Bakri et al., 2018). Secondly, carboxylic acids, hydroxy acids, keto acids, organic sulfonic acids and their derivatives existed in large quantities in soil, which could aggravate soil acidification, destroy the antioxidant defense system of plants, and inhibit plant growth (Liu et al., 2022; Zhao et al., 2022). In contrast, benzene and substituted derivatives, organonitrogen compounds and organooxygen compounds were organic compounds that were difficult to be absorbed by plants in the soils, and the degradation of these substances was conducive to the improvement of C, N and O cycles in the soil (Fallah et al., 2022; Jiang et al., 2022). It could be seen that the content of carboxylic acids, hydroxy acids, keto acids, organic sulfonic acids and their derivatives in tea rhizosphere soil decreased with the increase of soil pH, which effectively maintained the normal operation of antioxidant defense system of tea tree; secondly, the content of aryl halides, benzothiazoles, lactams, azolidines, pyridines and derivatives, steroids and steroid derivatives decreased in soil, which guaranteed the microecological balance of rhizosphere soil and facilitated the propagation of soil microorganisms. In contrast, imidazopyrimidines content decreased and its decomposition facilitated the increase of pyrimidines in the soil, which was conducive to the propagation of microorganisms in rhizosphere soil. In addition, it was found that with the increase of soil pH in tea rhizosphere, the content of benzene and substituted derivatives, organic compounds, organonitrogen compounds in rhizosphere soil which were not easy to decompose, decreased. It could be seen that with the increase of soil pH, the diversity and number of microorganisms in tea rhizosphere soil increased, and the cyclic capacity of C and N in the rhizosphere soil increased, and the resistance of tea tree increased, which in turn promoted the growth of tea tree.

## 4 Conclusion

**Figure 8:**
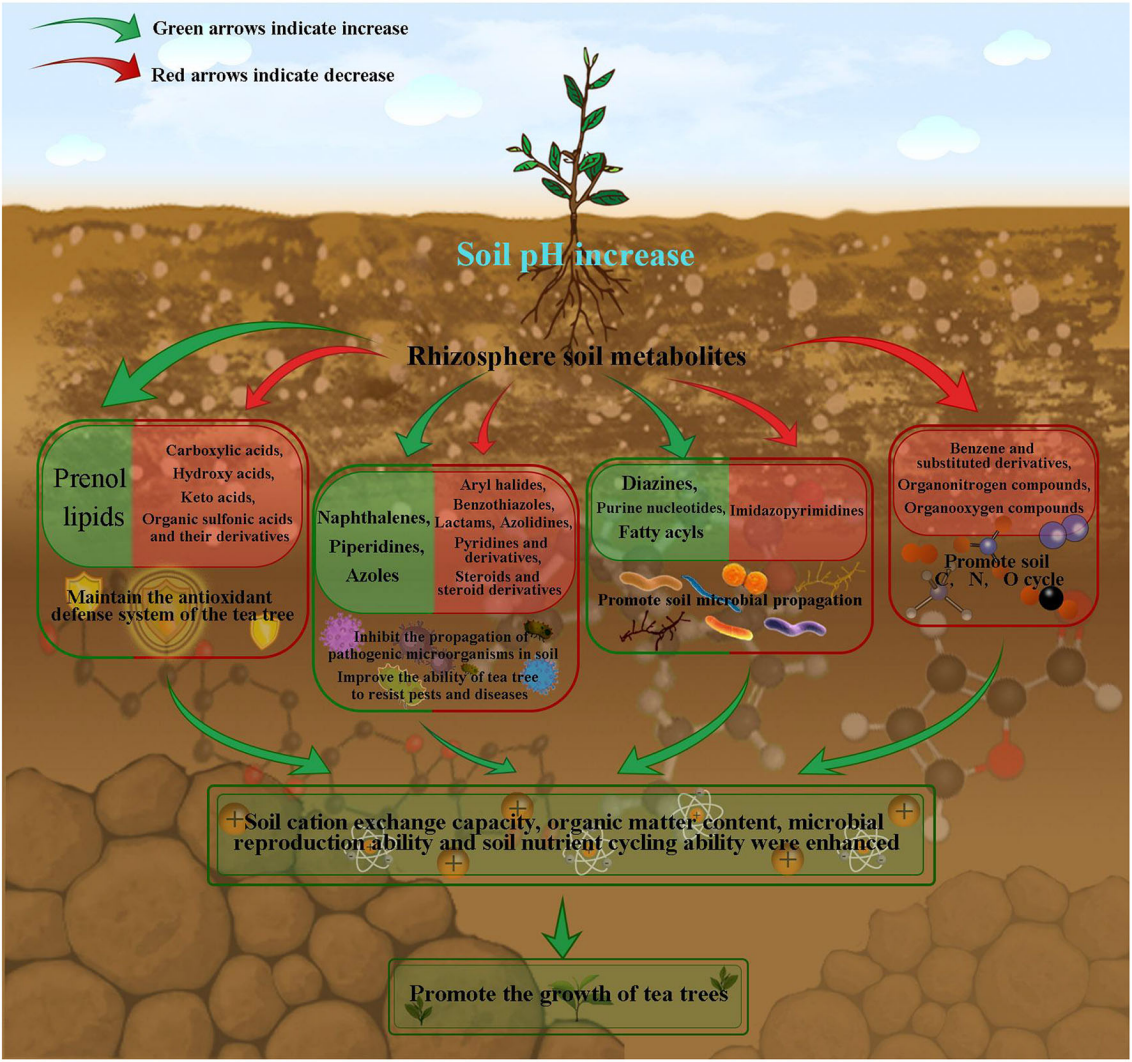
Mechanism simulation of effects of soil pH on metabolites in rhizosphere soil of tea tree.

In this study, we analyzed the soil physicochemical indexes, microbial indexes, metabolites and their relationships in tea rhizosphere with different pH values. The results showed (**Figure 8**) that with the increase of tea rhizosphere soil pH, the content of prenol lipids compounds increased, while the content of carboxylic acids, hydroxy acids, keto acids, organic sulfonic acids and their derivatives decreased, which effectively maintained the normal operation of antioxidant defense system of tea tree, enhanced the ability of tea tree to resist external environment stress, and improved the adaptability of tea tree to the environment. Secondly, the content of naphthalenes, piperidines and azoles compounds in tea tree rhizosphere soil increased, while the content of aryl halides, benzothiazoles, lactams, azolidines, pyridines and derivatives, steroids and steroid derivatives decreased, which reduced the number of pathogenic microorganisms and fungi in soil, guaranteed the microecological balance of rhizosphere soil, and improved the ability of tea trees to resist diseases and pests.

In addition, the content of compounds such as diazines, purine nucleotides, fatty acyls in tea rhizosphere soil increased, while the content of imidazopyrimidines decreased, which provided raw materials for the propagation of beneficial organisms and improved the diversity and number of microorganisms in tea rhizosphere soil. Secondly, in this study, as soil pH increased, microbial biomass carbon, microbial biomass nitrogen, microbial respiration intensity, bacterial number and actinomyces number of tea rhizosphere soil all showed an increasing trend, while the number of fungi showed a decreasing trend. This result also confirmed the conclusion of the above analysis of the characteristic metabolites in the rhizosphere soil of tea tree. At the same time, it was found in this study that with the increase of soil pH, the content of benzene and substituted derivatives, organonitrogen compounds and organooxygen compounds in rhizosphere soil, which were not easily utilized, decreased. However, it was also found in the study that soil organic matter and cation exchange capacity showed an increasing trend with the increase of soil pH. It could be seen that with the increase of soil pH, the diversity and number of microorganisms increased, and the cyclic capacity of C and N in tea rhizosphere soil increased, and the resistance of tea tree increased, which in turn promoted the growth of tea tree. In this study, we analyzed the effects of acidification on changes in microecosystem of tea tree rhizosphere soil from the perspective of soil metabonomics, and laid an important foundation for the remediation of acidified tea plantation soil.

## Data availability statement

The datasets presented in this study can be found in online repositories. The names of the repository/repositories and accession number(s) can be found in the article/**Supplementary Material**.

## Author contributions

JY and YuhW: Conceptualization, Visualization, Methodology, Writing – original draft, Formal analysis, Writing – review & editing, Funding acquisition. SL and YucW: Formal analysis, Writing – review & editing. PC and LH: Methodology, Investigation, Writing – original draft. XJ and JK: Methodology, Investigation. ZW and HW: Conceptualization, Visualization, Methodology, Writing – original draft, Formal analysis, Writing – review & editing, Funding acquisition. All authors contributed to the article and approved the submitted version.
